# Abdominal aortic aneurysm screening program using hand-held ultrasound in primary healthcare

**DOI:** 10.1371/journal.pone.0176877

**Published:** 2017-04-28

**Authors:** Antoni Sisó-Almirall, Belchin Kostov, Marta Navarro González, Daniel Cararach Salami, Alfonso Pérez Jiménez, Rosa Gilabert Solé, Concepció Bru Saumell, Lluís Donoso Bach, Mireia Villalta Martí, Luis González-de Paz, Rafael Ruiz Riera, Vicenç Riambau Alonso, Nihan Acar-Denizli, Marta Farré Almacellas, Manuel Ramos-Casals, Jaume Benavent Àreu

**Affiliations:** 1 Consorci d’Atenció Primària de Salut Barcelona Esquerra (CAPSBE), Grup Tranversal de Recerca en Atenció Primària, Institut d’Investigacions Biomèdiques August Pi i Sunyer (IDIBAPS), Barcelona, Spain; 2 Servei de Radiodiagnòstic, Centre de Diagnòstic per la Imatge, Hospital Clínic de Barcelona, IDIBAPS, Universitat de Barcelona, Barcelona, Spain; 3 Servei de Cirurgia Vascular, Institut Clínic del Tòrax, Hospital Clínic de Barcelona, Barcelona, Spain; 4 Department of Statistics, Faculty of Science and Letters, Mimar Sinan Fine Arts University, Istanbul, Turkey; 5 Institut Clínic de Medicina Interna i Dermatologia (ICMID), Hospital Clínic de Barcelona, Barcelona, Spain; Stellenbosch University Faculty of Medicine and Health Sciences, SOUTH AFRICA

## Abstract

We determined the feasibility of abdominal aortic aneurysm (AAA) screening program led by family physicians in public primary healthcare setting using hand-held ultrasound device. The potential study population was 11,214 men aged ≥ 60 years attended by three urban, public primary healthcare centers. Participants were recruited by randomly-selected telephone calls. Ultrasound examinations were performed by four trained family physicians with a hand-held ultrasound device (Vscan^®^). AAA observed were verified by confirmatory imaging using standard ultrasound or computed tomography. Cardiovascular risk factors were determined. The prevalence of AAA was computed as the sum of previously-known aneurysms, aneurysms detected by the screening program and model-based estimated undiagnosed aneurysms. We screened 1,010 men, with mean age of 71.3 (SD 6.9) years; 995 (98.5%) men had normal aortas and 15 (1.5%) had AAA on Vscan^®^. Eleven out of 14 AAA-cases (78.6%) had AAA on confirmatory imaging (one patient died). The total prevalence of AAA was 2.49% (95%CI 2.20 to 2.78). The median aortic diameter at diagnosis was 3.5 cm in screened patients and 4.7 cm (p<0.001) in patients in whom AAA was diagnosed incidentally. Multivariate logistic regression analysis identified coronary heart disease (OR = 4.6, 95%CI 1.3 to 15.9) as the independent factor with the highest odds ratio. A screening program led by trained family physicians using hand-held ultrasound was a feasible, safe and reliable tool for the early detection of AAA.

## Introduction

Abdominal aortic aneurysms (AAA) are dilatations of the aorta measuring ≥ 3 cm in diameter, commonly involving the infrarenal portion [1]. AAA are severe, silent, potentially life-threatening disorders. The estimated mortality associated with undetected or undiagnosed ruptured AAA is 50–80% [2]. Risk factors for AAA include age, male sex, smoking, hypertension, heart disease, family history of AAA, hypercholesterolemia and low HDL-cholesterol [3–11]. In the Norwegian Tromsø Cohort Study [4] the annual incidence of AAA was 0.4%. The prevalence is 4% in men aged 50–79 years and 7% in men aged 65–83 years. By contrast, in women aged 65–79 years the prevalence is < 1% [12–14]. AAA are often silent, leading to three major complications: AAA rupture, thrombi formation in the lumen, and compression of adjacent organs. Rupture, the most serious complication, correlates with the size of the AAA [15,16]. An additional risk factor for rupture is the rate of increase in the size of the AAA [14].

Ultrasonography is the gold standard tool for AAA screening due to its simplicity, safety, validity, cost-effectiveness, reproducibility and public acceptance, and is used in all studies that include screening programs.

Miniaturized ultrasound devices date back to the 1970s [17]. However, technological development over the last decade has revived interest in ultrasound devices, including small hand-held devices, with a view to new applications and bringing technology to the bedside [18]. Reduced size and cost and easier handling and transport mean that hand-held ultrasound may be a good complementary tool for family physicians.

In 2005, the U.S. Preventive Services Task Force recommended one-time AAA ultrasound screening in male ever-smokers aged 65–75 years. Recently, a systematic review of four population-based screening trials [19–22] by the U.S. Preventive Services Task Force concluded that screening of men aged ≥ 65 years reduced AAA-related mortality rates by 50% over 13–15 years [23]. Medicare data showed the utilization of AAA screening in the USA was under 1% in eligible patients [24]. However, in Spain, the health system is universal, public and free-at-the-point-of-use. We believe that a system with these characteristics (similar to the UK system) would have a higher rate of utilization of screening programs than those reported by studies in the USA. However, this hypothesis would require confirmation through a national screening campaign, which currently does not exist in Spain. There are no Spanish studies on the prevalence of AAA in primary healthcare (PHC), nor studies of hand-held ultrasound as a complementary tool.

The aim of this study was to assess the accuracy and reliability of a screening program led by PHC family physicians using a hand-held ultrasound device to determine the prevalence of AAA and associated cardiovascular diseases in a Mediterranean population.

## Materials and methods

### Study design

We carried out a prospective, interventional study in which participants were screened in PHC centers by family physicians using hand-held-ultrasound to diagnose AAA.

### AAA screening program

The prospective study population consisted of patients assigned to three urban, public PHC centers in Barcelona city (Catalonia, Spain) in June 2013. The inclusion criteria were male sex and age ≥ 60 years. The main difference in our study with respect to existing screening programs is the reduction from 65 years to 60 years of age. The main reason for this was to increase the years of life gained in patients with a possible AAA aged 60–64 years. Patients were recruited by randomly-selected telephone calls. One investigator called patients at random once a week to invite them to participate. Random selection was made by systematic sampling, considering an equal probability of selection for each patient. Self-referred patients were also accepted. Informative leaflets were made available in PHC center waiting rooms and entrances and physicians’ offices. In addition, a short video about the screening program was shown on televisions located in waiting rooms. Individuals interested in participating were contacted by PHC centers or their family physicians to schedule a hand-held ultrasound examination. We aimed to recruit the maximum number of participants (around 20 patients per week) during the study period (from June 2013 to October 2014) in clinical practice setting.

### Ultrasound examination

All ultrasound examinations were carried out in PHC centers by four family physicians (ASA,MNG,DCS,APJ) who received 25 hours of ultrasound training from two hospital radiologists (RGS,CBS). The training included theoretical training on the basic acquisition, obtention and interpretation of the images. All four physicians were then evaluated by the radiologists for their capacity to measure aortic diameters and to diagnose AAA.

**Fig 1:**
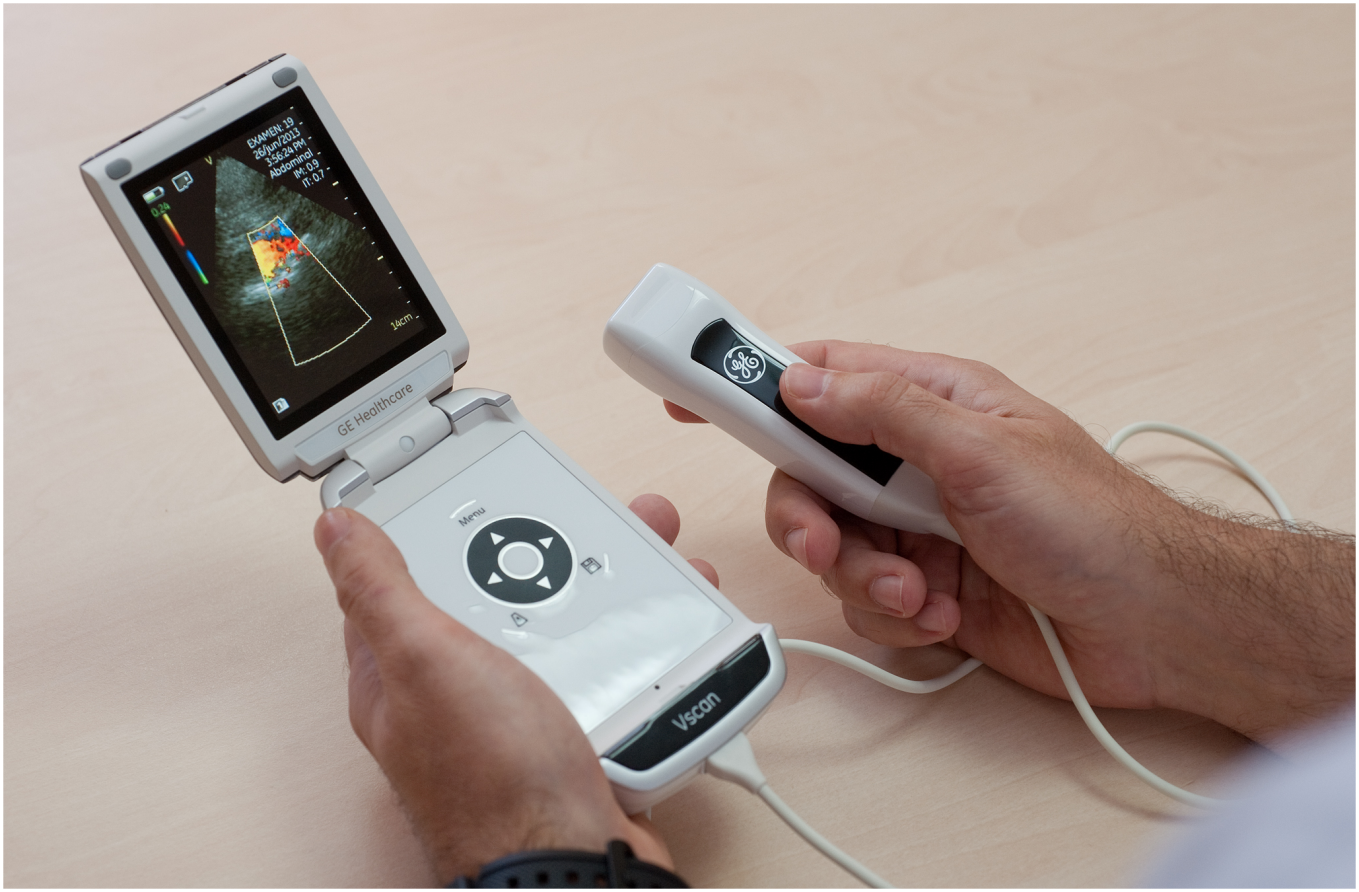
Vscan^®^ device.

Ultrasound examinations were performed with a hand-held ultrasound device (Vscan^®^, General Electric, USA). The device weighs 390 g and measures 135×73×28 mm, and has a screen size of 8.9 cm. It offers two-dimensional grey scale and live color Doppler imaging. The bandwidth ranges from 1.7 to 3.8 MHz and is adjusted automatically (Fig 1).

Aortic examinations were classified as complete or limited (due to technical difficulties, including excessive intestinal gas). Limited examinations, either due to a poor ultrasound window or to a large amount of air, were excluded from the final analysis. Standardized measurements were made using the external-to-external wall method [25] and aortas were visualized in three hard copy images: upper transverse projection of the abdominal aorta at the level of the epigastrium (celiac trunk), lower transverse section for distal view of aorta (pre-bifurcation) and longitudinal section (with origin of celiac trunk or superior mesenteric artery), and the maximum diameter in centimeters (cm) was determined. Aortas were classified in two groups according to size: normal (<3.0 cm) and aneurysmal (≥3.0 cm). Patients diagnosed with AAA were scheduled for confirmatory hospital imaging by standard ultrasound or computed tomography.

### Study outcomes

The primary outcome was to determine the feasibility of a PHC AAA screening program using hand-held ultrasound. Additional outcomes were: 1) to determine the risk factors associated with AAA, and 2) to estimate the total prevalence of AAA in males aged ≥ 60 years, computed as the sum of previously-known AAA recorded in the medical record, AAA detected by the screening program (validated by confirmatory imaging) and model-based, estimated, undiagnosed AAA.

### Study variables

Sociodemographic and biochemical variables were collected from the medical record in patients with a valid ultrasound measurement and included: age (years), body mass index (kg/m^2^), abdominal circumference (cm), systolic and diastolic blood pressure (mmHg), total cholesterol (mg/dl), high-density lipoprotein (mg/dl), low-density lipoprotein (mg/dl), triglyceride (mg/dl), creatinine (mg/dl), glycated hemoglobin (%) and estimated glomerular filtration rate by modified diet in renal disease (ml/min/m^2^).

Cardiovascular risk factors and cardiovascular diseases collected were obesity (body mass index>30 kg/m^2^), physical activity classified as sedentary lifestyle, moderate exercise, or intense exercise [26], hypertension, diabetes mellitus, hyperlipidemia, smoking habits, family history of AAA, chronic obstructive pulmonary disease, coronary heart disease, cerebrovascular disease and claudication. The Framingham-REGICOR index was used to assess the cardiovascular risk. This index is an adaptation of the Framingham coronary risk function to the characteristics of the Spanish population, has a well-contrasted calibration process [27], and classifies subjects as low (<5), moderate (5–9), high (10–14) and very high (≥15) cardiovascular risk.

Ultrasound examination variables collected included duration (in minutes), classification of the examination (complete/limited), aortic diameter at three points (xiphoid process, pre-bifurcation and longitudinal) and aorta classification (normal/aneurysmal).

In patients with a history of AAA, the aortic diameter, AAA repair, age at diagnosis and incidental detection were evaluated.

### Ethical aspects

This study was approved by the Hospital Clínic Clinical Research Ethics Committee (registration number 2011/6525). Patients who agreed to participate gave written informed consent. Study procedures complied with the Declaration of Helsinki regarding the conduct of biomedical research and respect for human rights. The study was registered at Clinical trials.gov (registration number NCT01882634).

### Statistical analysis

Demographic data were summarized using descriptive statistics. Categorical variables were expressed as absolute frequencies (%). Continuous variables were described as means [standard deviation (SD)] or medians [interquartile range (IQR)]. Continuous variables were analyzed using the Student's *t* test and categorical variables using the chi-square test and Fisher’s exact test. The odds ratios (OR) were calculated to study the association between cardiovascular risk factors and cardiovascular disease and AAA. Parameters with a p-value < 0.15 were included in the multivariate logistic regression analysis to determine the independent factors influencing AAA. The multivariate logistic regression model including risk factors independently associated with AAA was used to estimate the number and prevalence of undiagnosed AAA in non-screened patients fulfilling the inclusion criterion. The estimated number of undiagnosed cases of AAA combined with AAA cases diagnosed by the screening program and patients with a previous AAA yielded an overall expected prevalence [28]. An asymptotic method was used to compute the 95% confidence intervals (CI) of AAA prevalences. All significance tests were two-tailed, and values of p < 0.05 were considered significant. The statistical analysis was performed using the R statistics program version 3.3.2 for Windows [29].

## Results

### Population used for screening

**Fig 2:**
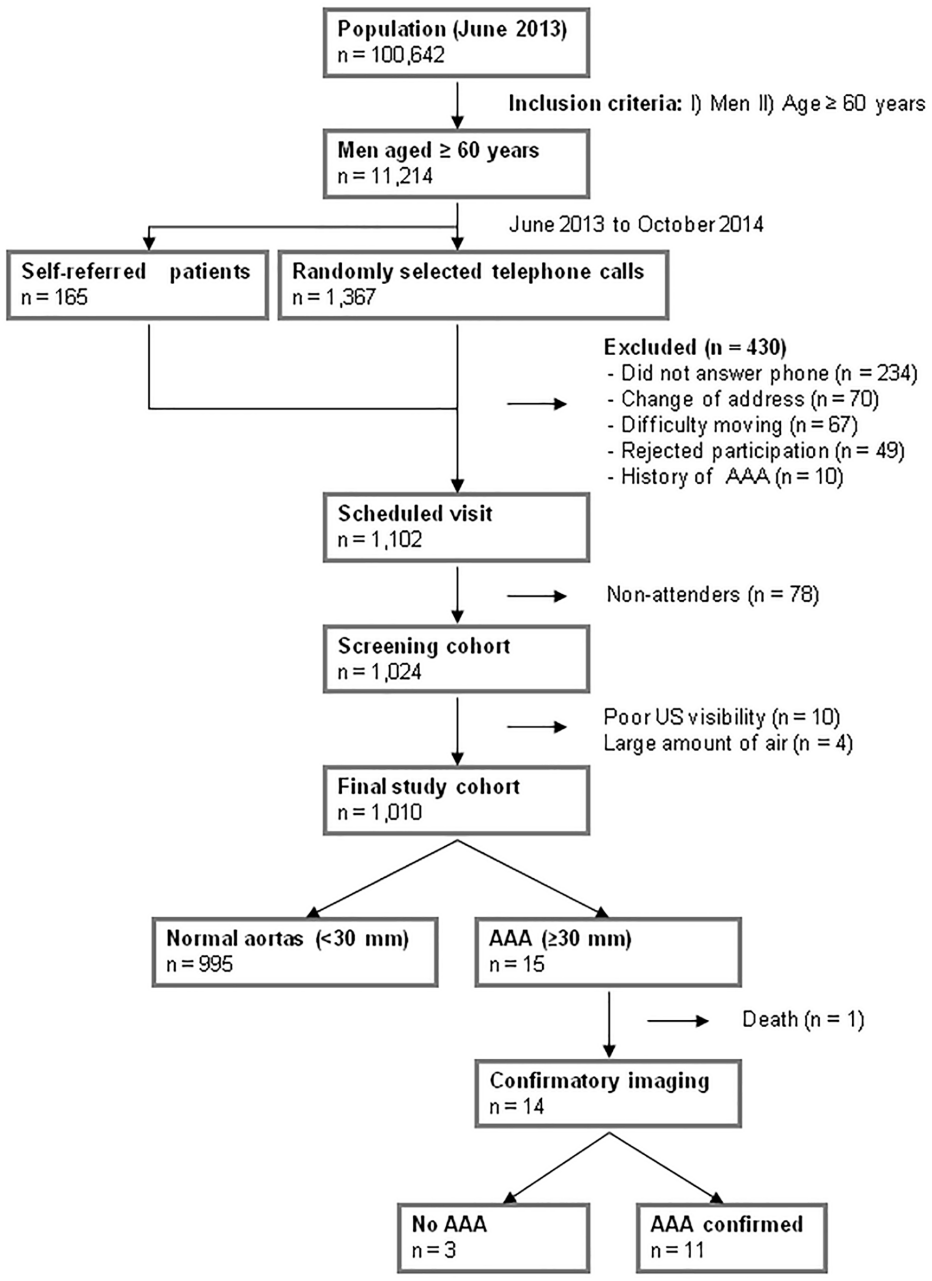
Screening flowchart.

Of 11,214 male patients aged ≥ 60 years, 1,367 (12.2%) were invited by randomly selected telephone calls, of which 420 were excluded (234 did not answer the phone, 70 had changed address, 67 had mobility problems, and 49 rejected participation). There were 165 self-referred patients, of whom 10 with a history of AAA were excluded. Therefore, 1,102 men had a scheduled visit for screening, of whom 1,024 (92.9%) attended. Ten examinations (1.0%) were excluded due to poor ultrasound visibility and 4 (0.4%) due to large amounts of air (limited examination). Thus, 1,010 men were finally studied (Fig 2).

### Characteristics of patients included in final study cohort

The mean age of the 1,010 men was 71.3 (SD 6.9) years, of which 794 (78.6%) patients were aged 65–84 years. The mean body mass index was 27.6 (SD 3.6) kg/m^2^, abdominal circumference 101.8 (SD 10.5) cm, systolic blood pressure 131.6 (SD 13.8) mmHg, total cholesterol 187.6 (SD 34.6) mg/dl and creatinine 1.1 (SD 0.2) mg/dl. Of 465 men with a glycated hemoglobin measurement, 429 (92.2%) had HbA1c < 8%: 99 (10.6%) patients out of 671 had a Modified Diet in Renal Disease < 60 ml/min/m^2^.

Cardiovascular risk factors are shown in Table 1. The prevalence of coronary heart disease was 13.6%, and 107 out of 648 patients (16.5%) had a high or very high predicted risk of a coronary event at 10 years according to the Framingham-REGICOR score.

**Table 1:** Cardiovascular risk factors and cardiovascular diseases of patients included in final study cohort.

Variable	Total (n = 1010)
Obesity (BMI>30 kg/m^2^)	241/993 (24.3)
Physical activity (n = 943)^¶^	
Sedentary lifestyle	147 (15.6)
Moderate	507 (53.8)
Intense	289 (30.6)
Hypertension	663 (65.6)
Diabetes mellitus	275 (27.2)
Hyperlipidemia	547 (54.2)
Current smoker	143 (14.2)
Ever smoked	665 (65.8)
Family history of AAA	13 (1.3)
COPD	77 (7.6)
Coronary heart disease	137 (13.6)
Cerebrovascular disease	38 (3.8)
Claudication	42 (4.2)
Renal disease*	99/671 (10.6)
REGICOR risk score (n = 648)	6.8 ± 3.6
Low (<5)	226 (34.9)
Moderate (5–9)	315 (48.6)
High (10–14)	83 (12.8)
Very high (≥15)	24 (3.7)

Values are shown as mean ± SD or frequency (%).

BMI: Body mass index; COPD: Chronic obstructive pulmonary disease.

^¶^ Intense: lifting heavy objects, digging, aerobics, or fast bicycling; moderate: carrying light loads, bicycling at a regular pace, or playing tennis doubles; sedentary lifestyle: regular physical activity does not involve activities from the other categories, time sitting at work, at home, studying, and at leisure.

* Estimated Glomerular Filtration Rate by MDRD (Modified Diet in Renal Disease) < 60 ml/min/m^2^.

### Ultrasound examination results and confirmatory imaging

**Fig 3:**
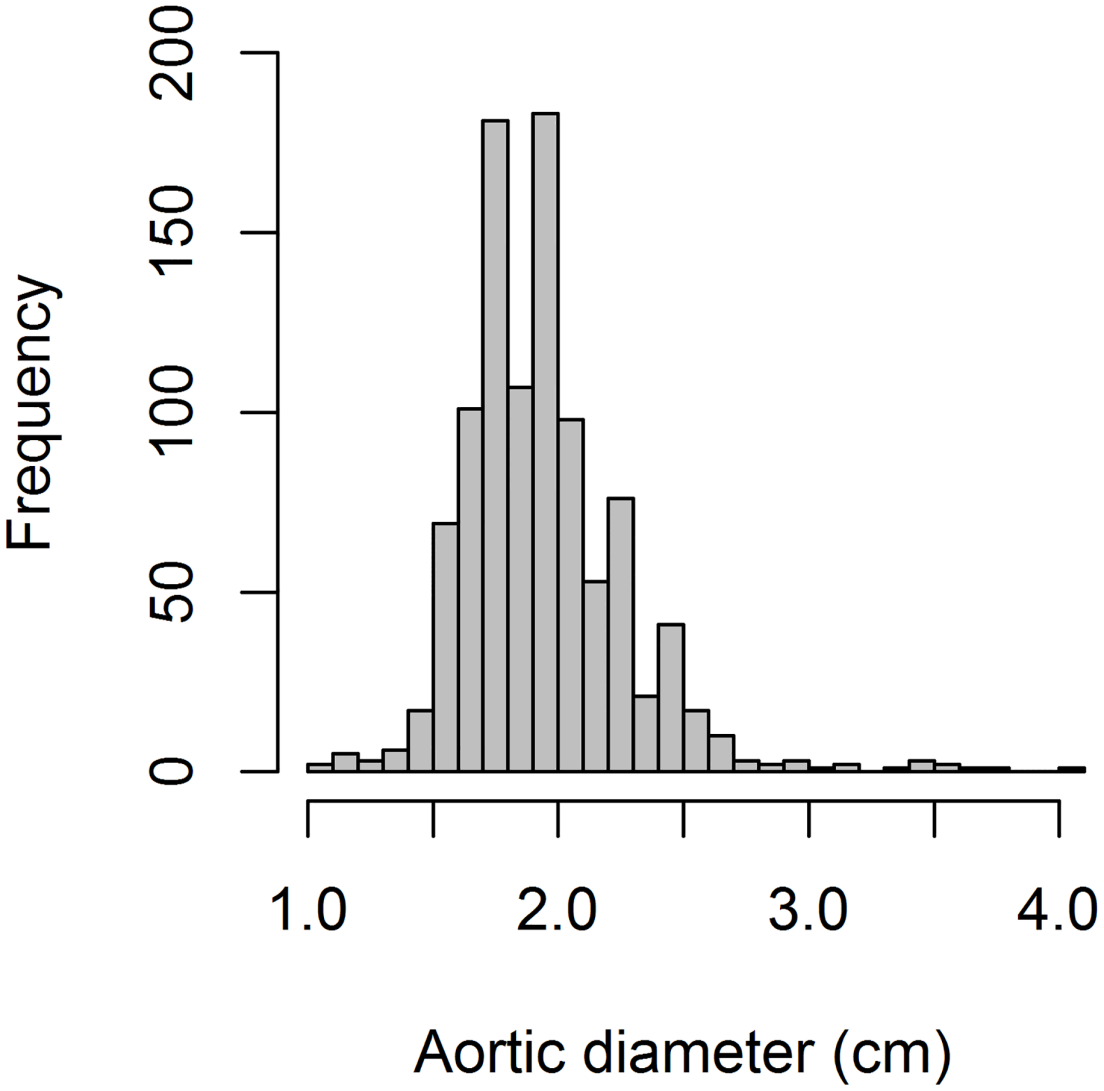
Distribution of aortic diameters.

The median examination time was 4 [IQR 3–5] minutes. Median aortic diameters were 1.8 [IQR 1.7–2.1] cm (xiphoid process), 1.7 [IQR 1.6–1.9] cm (pre-bifurcation) and 1.8 [IQR 1.7–2.0] cm (longitudinal). With respect to size (Fig 3), 995 (98.5%) men had normal aortas (aortic diameter <3.0 cm) and 15 (1.5%) had AAA (aortic diameter ≥ 3.0 cm).

Patients with AAA were aged between 66 and 85 years. The median aortic diameter at diagnosis was 3.5 cm [IQR 3.2–3.6]. The AAA diameter was 3.0 to 3.5 cm in 10 patients (66.7%) and 3.6 to 4.1 cm in 5 (33.3%). In all AAA cases, the diagnosis was verified by standard ultrasound or computed tomography, except for one patient who died before confirmatory imaging. Age, aortic size, risk factors and confirmatory imaging of patients with AAA are shown in Table 2. Of the 14 remaining patients with AAA on ultrasound, the AAA was confirmed in 11 (78.6%).

**Table 2:** Patients with AAA on ultrasound examination with confirmatory imaging, ordered from smallest to largest size.

Case	Age	Aorta size (Vscan^®^)	Risk factors	Follow-up imaging	Size follow-up
1	75	3.0	Ex-smoker, HTA, HLD, COPD, CD	Ultrasound	3.1
2	70	3.0	HTA, HLD, CHD	Computed tomography	3.0
3	68	3.0	Ex-smoker, HLD	Ultrasound	3.2
4	75	3.1	Ex-smoker, HLD, CHD	Ultrasound	2.6
5	68	3.2	HLD	Computed tomography	Normal aorta (<3.0)^¶^ ^†^
6	71	3.2	-	Computed tomography	Normal aorta (<3.0)^¶^
7	69	3.4	Ex-smoker, HTA, HLD	Computed tomography	3.6
8	85	3.5	Ex-smoker, HTA, HLD, CHD	No follow-up imaging*	-
9	80	3.5	Ex-smoker	Computed tomography	3.5
10	72	3.5	Ex-smoker, HTA, DM, HLD, CHD	Computed tomography	3.6
11	76	3.6	Smoker, HTA, HLD, COPD, CHD, Claud.	Computed tomography	3.6
12	73	3.6	Ex-smoker, HTA	Ultrasound	3.6
13	69	3.7	Ex-smoker, DM, HLD	Ultrasound	3.7
14	79	3.8	Ex-smoker, DM, HLD, COPD, CHD	Computed tomography	3.4^†^
15	66	4.1	Ex-smoker, HTA, HLD, CHD	Ultrasound	4.2

* Patient died before confirmatory imaging.

^¶^ Size not mentioned.

^†^ Luminal thrombus on the computed tomography image.

HLD: Hyperlipidemia; HTA: Hypertension; COPD: Chronic obstructive pulmonary disease;

CD: Cerebrovascular disease; Claud.: Claudication; CHD: Coronary heart disease; DM: Diabetes mellitus

### Risk factors associated with AAA

The association between AAA and risk factors is shown in Table 3. Ever smoking (10/11), hyperlipidemia (9/11) and coronary heart disease (5/11) were the most prevalent risk factors associated with AAA. Chronic obstructive pulmonary disease was less prevalent (3/11) but was also significantly associated with AAA. Multivariate logistic regression analysis identified coronary heart disease (OR = 4.6, 95%CI 1.3 to 15.9) as an independent risk factor associated with AAA. The most prevalent risk factor, ever smoking, was closely associated with AAA (OR = 4.3, 95%CI 0.8 to 80.5) but was not significant due to the small numbers of patients with AAA.

**Table 3:** Comparison of risk factors associated with AAA.

Risk Factor	No AAA (n = 998)	AAA* (n = 11)	*P*^†^	OR [95%CI]^¶^
Obesity (BMI>30 kg/m^2^)	237/981 (24.2)	4 (36.4)	0.313	
Physical activity			0.640	
Sedentary lifestyle	146/931 (15.7)	1 (9.0)		
Moderate	501/931 (53.8)	5 (45.5)		
Intense	284/931 (30.5)	5 (45.5)		
Hypertension	655 (65.6)	7 (63.6)	1	
Diabetes mellitus	272 (27.3)	3 (27.3)	1	
Hyperlipidemia	537 (53.8)	9 (81.8)	0.074	2.7 [0.7–18.0]
Current smoker	142 (14.2)	1 (9.1)	1	
Ever smoker	654 (65.5)	10 (90.9)	0.110	4.3 [0.8–80.5]
Family history of AAA	13 (1.3)	0 (0)	1	
COPD	74 (7.4)	3 (27.3)	0.045	3.3 [0.7–12.1]
Coronary heart disease	131 (13.1)	5 (45.5)	0.010	4.6 [1.3–15.9]
Cerebrovascular disease	37 (3.7)	1 (9.1)	0.346	
Claudication	41 (4.1)	1 (9.1)	0.375	
Renal disease	98/662 (14.8)	1/8 (12.5)	1	
REGICOR risk score			0.557	
Low (<5)	224/641 (34.9)	2/7 (28.6)		
Moderate (5–9)	312/641 (48.7)	3/7 (42.8)		
High (10–14)	81/641 (12.6)	2/7 (28.6)		
Very high (> = 15)	24/641 (3.8)	0 (0)		

Values are shown as mean ± SD or frequency (%)

BMI: Body mass index; COPD: Chronic obstructive pulmonary disease

^†^ Percentages were compared using uncorrected χ^2^ test or Fisher’s exact test

^¶^ Multivariate logistic regression analysis of variables associated with AAA.

### Clinical evolution of patients with a history of AAA and prevalence of AAA

During the study period, 12 AAA cases were identified among non-screened men. These 12 patients with AAA combined with the 160 with a history of AAA yielded a prevalence of 1.7% (95%CI 1.5 to 1.9) of AAA diagnosed without screening. The mean age of these patients at diagnosis was 70.8 (SD 7.3) years. Of the 172 patients, 110 were incidentally detected (64%), and 89 (51.7%) had a history of AAA repair. Median time from diagnosis to surgery was 145 [IQR 20.8–719.8] days. At diagnosis, the median aortic diameter in non-screened patients with AAA was 4.7 [IQR 3.7–5.5] cm, greater than the median aortic diameter at diagnosis in screened patients (p<0.001).

Model-based estimates showed 96 undiagnosed AAA in non-screened men, with an estimated prevalence of around 0.93% (95%CI 0.74 to 1.12). The sum of previously known AAA, AAA detected by the screening program and model-based estimated undiagnosed AAA was 279 patients. Therefore, the overall estimated prevalence of AAA in men aged ≥ 60 years was 2.49% (95%CI 2.20 to 2.78) in the Spanish study population.

## Discussion

This is the first Spanish study of a PHC screening program using hand-held ultrasound. The estimated overall prevalence of AAA was 2.49% in men aged ≥ 60 years. Only one study, in Western Australia, has analyzed the prevalence of AAA according to place of birth [30]: men born in Scotland had a very high age-adjusted prevalence of AAA, while those of Mediterranean origin, principally from Italy, Greece and the former Yugoslavia, had a distinctly lower prevalence with respect to Australian-born men. Studies in Chichester, UK [20], the MASS, UK study [31,32], Viborg, Denmark [21,33], Italy [34], The Netherlands [35] and Western Australia [22,30] found the prevalence of AAA in population-based studies ranged from 4.0% to 7.2%. However, a Swedish study of population-based screening of >22,000 subjects reported a prevalence of 2.2%, the lowest estimate to date [36] and suggested that the current target population differed from those included in previous studies of AAA prevalence in men aged ≥ 65 years. Because smoking was the risk factor most strongly associated with AAA [37], changes in smoking habits could explain this lower-than-expected prevalence, and our results support this argument: we found an active smoking rate of 14%, similar to the 13% found in the Swedish study.

Patients in our screening program had a median aortic diameter of 3.5 [IQR 3.2–3.6] cm compared with 4.7 [IQR 3.7–5.5] cm (p<0.001) in patients diagnosed incidentally. The probability of a ruptured aneurysm is directly proportional to size, and a significant difference of 12 mm may be a very strong argument in favor of a PHC AAA screening program to avoid a large number of patients with ruptured AAA in whom a prior opportunity for detection is missed [38].

Hand-held ultrasound is getting increasing interest in various medical fields and strata [39]. Bonnafy et al [40] found that abdominal aortic measurements performed by trained medical students were similar to those obtained by experts. Durham [41] describes three situations where hand-held ultrasound administered by non-experts has saved time and had clinical benefits: ectopic pregnancy, AAA, and pericardial effusion. Andersen et al [42], using Vscan^®^, found that sensitivity, specificity and predictive values were 100%, in agreement with our previous validation study [43]. Dijos et al [44] found that hand-held-ultrasound with Vscan^®^ for AAA screening had a diagnostic accuracy and abdominal aorta measurement comparable to conventional ultrasound (Pearson’s correlation = 98%). The false positive rate of 21.4% (3/14) found in the current study could be considered high. However, it should be noted that one of these patients had aortic ectasia (patient #4) and another had a luminal thrombus (patient #5). Thus, it could be argued that these are not truly false positives and that the sensitivity is, therefore, higher. Moreover, there is no consensus on the best methods of measuring the diameter of the abdominal aorta [25]. The inner-to-inner and the leading edge-to-leading edge methods give smaller measures of the aortic diameter than the external-to-external wall method, with the estimated prevalence varying from -22% (inner-to-inner) to +36% (external-to-external wall) depending on the method. We chose the external-to-external wall method because confirmatory hospital imaging by standard ultrasound or computer tomography was carried out only to verify the diagnosis. In other words, we accepted an increase in the rate of false positives in order to minimize the risk of false negatives, which could not be observed. This study was performed under real-life clinical setting. Confirmatory hospital imaging by standard ultrasound or computer tomography was used only to verify the diagnosis. Therefore, the study design did not allow some measurements, such as the specificity, negative predictive value or false negative rate. However, this was not the aim of the study and several reports have already demonstrated that the Vscan^®^ has a good diagnostic accuracy for AAA screening [43,44].

Although we expanded the age range downwards to ≥ 60 years, the prevalence of AAA was very similar to that observed in studies in the general population. A Cochrane systematic review [45] of AAA screening that included 127,981 men and 9,342 women concluded that screening significantly reduced direct mortality from AAA in men (OR = 0.60, 95%CI 0.47 to 0.78) but not in women (OR = 1.49, 95%CI 0.25 to 8.94), and, in men, there was a significant reduction in the incidence of ruptured AAA (OR = 0.45, 95%CI 0.21 to 0.99). Other agencies have made similar recommendations, but differ in the inclusion criteria and the age range included: in 2006 the American College of Cardiology and American Heart Association recommended AAA screening in men aged 65–75 years or former smokers aged ≥ 60 years with first-degree relatives with AAA [46]; the Society for Vascular Surgery and Society for Vascular Medicine and Biology recommends ultrasound screening for all men aged 60–85 years and all individuals aged > 50 years with a first-degree family history of AAA [47,48]. Although most clinical trials have used 65 years as the age of onset of screening, the optimal age remains unclear. Therefore, we extended the age range downwards to 60 years with the aim of detecting younger patients and increasing the life-years gained. Some studies have evaluated the age of 60 years as the cut-off [49], such the EVAR trial (United Kingdom EndoVascular Aneurysm Repair) which compared endovascular surgery versus open repair of AAA in patients aged ≥ 60 years [50]. If subjects aged 60–64 years were excluded from our study, the estimated prevalence of AAA in men aged ≥ 65 years would be 2.89% (95%CI 2.54 to 3.24). The prevalence of AAA in men aged ≥ 65 years with a history of tobacco use or family history of AAA was 7.3% (95%CI 5.6 to 9.0).

With respected to the reduction in mortality, in a Danish study application of a screening program meant 107 years of life gained after 10 years in screened patients. The relative risk associated with screening was highly favorable to the intervention group, with a reduction in AAA-attributable mortality (RR = 0.33, 95%CI 0.16 to 0.71), fewer AAA ruptures (RR = 0.27, 95%CI 0.13 to 0.60) and fewer urgent surgeries (RR = 0.25, 95%CI 0.09 to 0.66). The study concluded that AAA screening in Danish men aged 64–73 years reduced AAA-specific mortality by 67%, and the number of patients needed to be included in a screening program to save one life was 352 patients [33,51]. Public health measures could further reduce global AAA mortality, with the greatest benefits occurring in younger patients [52].

Despite the safety of ultrasound, its use is not widespread in PHC for AAA screening. Although several intervention strategies have been proposed in primary care to improve compliance with AAA screening [53], ultrasound is still little used compared with other settings [54]. AAA screening using a hand-held ultrasound device was faster for early detection: our study required only 4 minutes per patient. Moreover, it would be cheaper than traditional AAA screening programs that have a modest effect on AAA rupture or all-cause mortality [55,56] with an estimated cost per patient of $53 [57]. Some recent opinions suggest ultra sound should be brought to the point of patient care, and should be in the black bag of every general practitioner [58,59], or should form part of the periodic physical examination [60]. In 2013, the UK National Health Service (NHS) introduced universal AAA screening in men aged ≥ 65 years. All men are invited for screening during the year they turn 65 while previously-unscreened men aged > 65 years can self-refer for screening by contacting their local screening service [61]. A similar program should be introduced in Spain.

Our study had some limitations. The combination of self-referred men and men chosen randomly could potentially lead to considerable heterogeneity in the cohort. In our study, most of the 1024 participants screened were randomly selected (85%). In fact, random selection was considered to avoid selection bias (and include patients who rarely visit primary healthcare centers). Self-referred patients were also accepted for ethical reasons. Thus, in our opinion, the heterogeneity of our cohort was not meaningful. Although ultrasound is the gold-standard for the detection of AAA, obese patients generally present a worse acoustic window, making it more difficult to visualize an aorta that is located in a much deeper plane. The solution could be technological: in populations with a high prevalence of obesity, technologically- improved ultrasound machines with a larger screen size are necessary. In fact, in our study, the mean BMI in patients with a non-diagnostic study was 35.8 kg/m^2^. We analyzed a smaller sample size than other studies, detecting 11 cases of AAA. Although our findings are similar to those of other reports with larger sample sizes, the results of the multivariate model for the assessment of risk factors and the estimate of the expected prevalence should be interpreted with caution. In the 11 patients aged ≥ 65 years in whom small AAA (all between 30 to 42 mm) were detected, follow up with a new abdominal ultrasound at 12 months was necessary. However, the present study only shows the results of the first exploratory ultrasound. Larger studies should be conducted to improve recruitment strategies and increase the number of participants.

In conclusion, this prospective study of a screening program for AAA using Vscan^®^ led by family physicians in PHC centers suggests that the program was easy to administer, rapid, and successful in the early detection of AAA. Hand-held ultrasound could be a feasible tool for the PHC family physician, as it is easily repeatable and safe, without a risk of radiation. In the near future, technological advances may further improve the portability, reliability, and accuracy of hand-held ultrasound devices.

## Supporting information

S1 File Dataset. (XLSX)
